# Mature Cystic Teratoma of the Fallopian Tube in a Postmenopausal Woman: A Case Report and Review of the Literature

**DOI:** 10.1155/2015/583021

**Published:** 2015-01-15

**Authors:** Mustafa Erkan Sari, Ozhan Ozdemir, Pinar Kadirogullari, Funda Arpaci Ertugrul, Cemal Resat Atalay

**Affiliations:** Department of Obstetrics and Gynecology, Ankara Numune Education and Research Hospital, 06010 Ankara, Turkey

## Abstract

*Background*. Mature cystic teratomas of the fallopian tube are extremely rare and only 54 cases have been reported in the literature. In this paper, we report a mature cystic teratoma of the fallopian tube in a postmenopausal woman and we report the review of literature of tubal cystic teratomas. *Case*. A 62-year-old, gravida 4 postmenopausal woman presented with pain in the right lower abdominal region for a long time. An 88 × 72 × 95 mm heterogeneous mass which contained calcifications and lipoid components was detected in the right adnexal region by transvaginal ultrasonogram (TV-USG). Serum tumour markers, namely, CA125, CA15-3, and CA19-9, were within normal range. A laparotomy revealed a 9 × 10 cm cystic mass within the fimbrial region in the right fallopian tube, and right salpingoopherectomy was performed consequently. Microscopic examination revealed squamous epithelium with sebaceous glands and hair follicles, and pseudostratified ciliated respiratory epithelium with cartilage and mucous glands. Because the frozen section resulted in a benign dermoid cyst, no further operative procedure was performed. The postoperative follow-up was uneventful and the patient was discharged on the second postoperative day. *Conclusion*. In cases of undetermined pelvic or abdominal masses, a teratoma of the fallopian tube should be considered.

## 1. Introduction

Mature cystic teratomas, also known as dermoid cysts, originate from primordial germ cells and are composed of well-differentiated derivatives of any combination of three germ layers: ectoderm, mesoderm, and endoderm [1]. Although mature cystic teratoma is the most common benign ovarian neoplasm, its occurrence in the fallopian tube is very uncommon. Since the first report in 1865, there have been over 70 cases of teratomas of the fallopian tube [2]. Herein, we report a mature cystic teratoma of the fallopian tube in a postmenopausal woman who was operated on due to an adnexal mass and we reviewed the literature of tubal teratomas.

## 2. Case Report 

**Figure 1:**
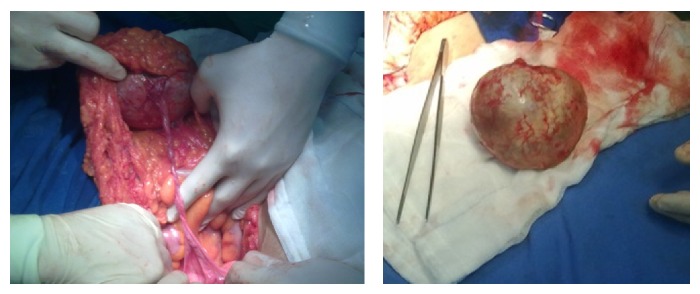
Mature cystic teratoma arising from the fimbrial end of the right fallopian tube.

**Figure 2:**
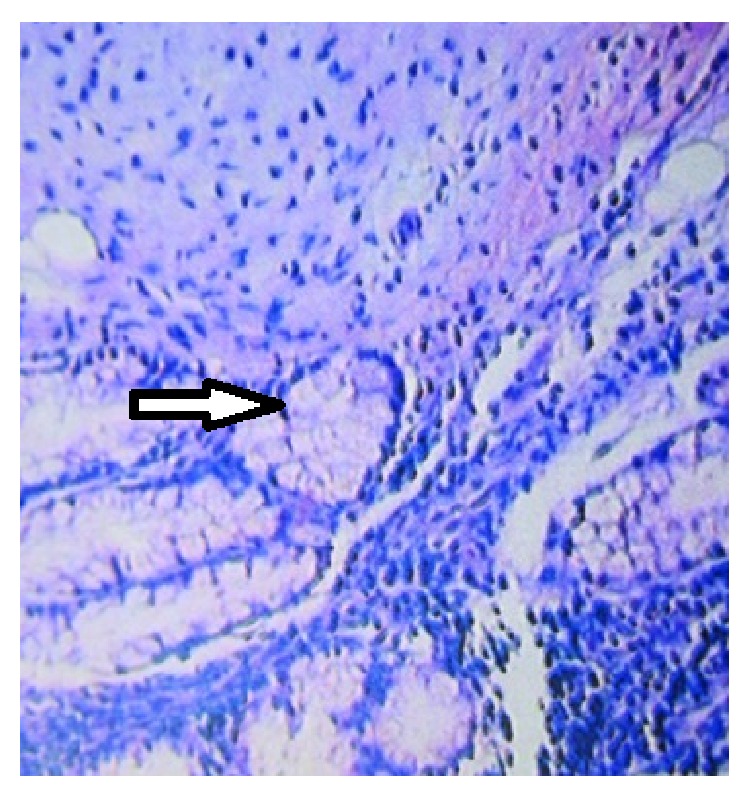
Microscopic observations of the mature teratoma demonstrate a cyst lined entirely by well-differentiated keratin-producing squamous epithelium (magnification, ×100).

A 62-year-old gravida 4 para 4 postmenopausal patient applied to our clinic with sustained bilateral pelvic pain. She had a history of two laparotomies. In the transvaginal ultrasonogram (TV-USG), we detected an 88 × 72 × 95 mm heterogeneous mass in the right adnexal region which contained calcifications and lipoid components. Her uterus was atrophic, and the endometrium was irregular. The endometrial biopsy (performed for irregular endometrium) and cervical smear results were normal as well as the routine laboratory tests and previously reported tumour markers. The laparotomy revealed an atrophic right ovary and a cystic mass located in the fimbrial portion of the right fallopian tube (Figure 1). Consequently, right salpingoophorectomy was performed, and a 9 × 10 cm cystic mass was observed within the fimbrial region which was filled with whitish gelatinous and yellowish greasy material with hairs. Because the frozen section result was a benign dermoid cyst, no further operative procedure was performed. Microscopic examination revealed squamous epithelium with sebaceous glands and hair follicles and pseudostratified ciliated respiratory epithelium with cartilage and mucous glands (Figure 2). The postoperative follow-up was uneventful and the patient was discharged on the second postoperative day.

## 3. Discussion

Teratomas are classified as mature teratomas (cystic or solid), immature teratomas, and highly specialized teratomas such as struma ovarii. Mature cystic teratomas, also known as dermoid cysts, are the most common type with a peak incidence at ages 20–40. These tumours are usually unilocular but they may be multilocular and may contain various tissues such as hair, skin, teeth, sebaceous material, cartilage, bone, salivary glands, and nerve tissue at different ratios [1].

Although teratomas are the most common benign ovarian neoplasms, their occurrence in the fallopian tube is very uncommon. The first 25 cases of tubal teratomas were collected by Aaron in 1941 [3]. After that, Mazzarella et al. reviewed the literature once again and made the most comprehensive review of tubal teratomas, both solid and cystic, by reporting a total of 44 cases [4]. Khatib et al. reported a review of 73 tubal teratoma cases worldwide [5]. Up to date, approximately 75 cases of tubal teratomas have been reported in the English literature, and among these, 54 cases were cystic teratomas [6].

The pathogenesis of teratomas has not clearly been understood; however they are believed to arise from germ cells migrating from the yolk sac to the primitive gonadal bud. Tubal teratomas may result from the failure of these germ cells to reach the ovaries [4]. They are usually attached to the tubal mucosa by a pedicle and are commonly located in the ampulla or the isthmus [2]. These tumors are usually asymptomatic but they may sometimes lead to reduced parity, menstrual irregularity, leukorrhea, postmenopausal bleeding, or abdominal pain.

In this paper, we report a mature cystic teratoma of the fallopian tube in a postmenopausal woman who was operated on due to an adnexal mass, and we reviewed the 54 cystic teratoma cases in the literature. In Table 1, we summarized 30 cases of cystic teratoma of the fallopian tube with all characteristics (patient's age, gravidity and parity, tumor size, and location) which were reported in related studies included. Sixty percent of the patients were diagnosed at the 3rd and 4th decades of age, and 68% of them had two or fewer gravidities. The tumor sizes of cystic tubal teratomas have been reported to range between 0.5 cm and 17 cm. Only two cystic teratoma cases were bilateral, and two cases were postmenopausal [7]. Ectopic pregnancy was detected in four cases. One mature cystic teratoma case was detected during a caesarean section in a term pregnant woman [8]. Tubal teratoma may be accompanied by ectopic pregnancy, uterine leiomyomatosis, uterine malformation, and ovarian cysts such as struma ovarii or other neoplasms including endometrial adenocarcinoma [6]. Our case was 62 years old with a history of four parities, and the cystic teratoma was located at the fimbrial portion of the right fallopian tube with a size of 9 × 10 cm.

Most tubal mature teratomas are found incidentally at routine control or during pelvic surgery, and tubal teratomas are often misdiagnosed as ovarian teratomas during physical examination prior to surgery, similar to our case. When a pelvic mass which is considered to be a teratoma is noticed, the probability of tubal teratoma should immediately be considered. Although the incidence of tubal teratomas is low, awareness of its presence is necessary. Particularly, pathologists must consider the possibility of a tubal teratoma when the origin of the adnexal mass is grossly ambiguous, because tubal teratomas are often misdiagnosed as ovarian teratomas in radiologic studies [9]. Due to the risk of malignancy, pathologists should be careful during the microscopic examinations of the specimen.

## Figures and Tables

**Table 1:** Cystic teratomas of the fallopian tube (the cases in which all characteristics were noted in the literature).

First author	Age	Gravidity/parity	Tumor size (cm)	Localization	Other
Orthmann (1914) [10]	33	G1	9 × 3 × 4	R, ampulla	None
Ohno (1918) [11]	36	G1	6.5 × 5.7 × 4.5	R, isthmic	None
Delannoy (1923) [12]	32	G0	15 × 5 × 13 (R) 15.5 × 5.5 × 12 (L)	Bilateral, third portion	None
Neumann (1927) [13]	36	G0	3.4 × 1	L, ampulla	Ectopic pregnancy
Leicher (1951) [14]	24	G0	17 × 18	L, ampulla	Eversion and elephantoid mucosal edema
Shirley (1954) [15]	33	G1P1	9 × 7 × 6.5	L, ampulla	None
Zelinger (1960) [16]	30	G2P2	1 × 0.5	L, isthmus	Ruptured ectopic pregnancy
Legerlotz (1964) [17]	25	G1	10 × 8	R, isthmus	None
Gray (1969) [18]	51	G2P2	1.5 × 1.5	R, isthmus	None
Lorenz (1972) [7]	36	G1P1	9 × 10 × 7 (R), 4 × 3.5 (L)	Bilateral	None
Hurd (1978) [19]	31	G0	7.5 × 7.5 × 6	R, middle	None
Crone (1981) [20]	39	G3P3	5 × 5	R, ampulla	None
Horn (1983) [21]	30	G3P3	0.8 × 0.5	R, ampulla	None
Bouquet de Joliniere (1986) [22]	35	G3P1	2 × 2	R, isthmus	None
Massouda (1988) [23]	24	G0	2.8 × 1	R, isthmus	Ectopic pregnancy
Frost (1989) [24]	30	G8P6	10 × 10 × 6	L, ampulla	None
Kutteh (1991) [25]	25	G3P3	2 × 2	L, isthmus	Ruptured ectopic pregnancy
Lai (1993) [26]	33	G0	5 × 2.5 × 2	R, ampulla	None
Pai (1997) [27]	30	G6P3	1 × 1	L, isthmus	None
Hseih (1998) [8]	36	G2p1	2.5 × 2 × 2	L, middle portion	Term pregnancy, emergency caesarean
Matts (1998) [28]	17	G0	12 × 12 × 8	R, ampulla	None
Amina (2005) [29]	35	G1P1	2 × 2	L, ampulla	None
Haslík (2006) [30]	24	G0	12 × 5	L, ampulla	Torsion of the left adnexa
Johnson (2006) [31]	31	G0	2 × 1.5	L, ampulla	None
Fujiwara (2010) [32]	31	G0	2 × 1.5	R, ampulla	None
Sung (2011) [9]	44	G4P1	3.3	R, ampulla	None
Khatib (2013) [5]	50	G2P2	3 × 3	R, ampulla	None
Li (2013) [6]	23	G0	8 × 4	L, ampulla	Incomplete uterine mediastinum
Yao (2014) [2]	30	G0	0.6	L, ampulla	None
**Our case (2014)**	**62**	**G4P4**	**7 × 9 × 9.5**	**R, ampulla**	**None**
